# Study of Fluorinated Quantum Dots-Protein Interactions at the Oil/Water Interface by Interfacial Surface Tension Changes

**DOI:** 10.3390/ma11050750

**Published:** 2018-05-08

**Authors:** Carolina Carrillo-Carrión, Marta Gallego, Wolfgang J. Parak, Mónica Carril

**Affiliations:** 1 Centro Singular de Investigación en Química Biológica y Materiales Moleculares (CiQUS) y Departamento de Física de Partículas, Universidad de Santiago de Compostela, 15782 Santiago de Compostela, Spain; 2 Bioengineered Particles Group, CIC BiomaGUNE, 20014 San Sebastian, Spain; mgallego@cicbiomagune.es (M.G.); wolfgang.parak@uni-hamburg.de (W.J.P.); mcarril@cicbiomagune.es (M.C.); 3 Fachbereich Physik and CHyN, Universität Hamburg, 22607 Hamburg, Germany; 4 Ikerbasque, Basque Foundation for Science, 48011 Bilbao, Spain

**Keywords:** interfacial tension, quantum dots, fluorine, protein-nanoparticle interaction, protein corona

## Abstract

Understanding the interaction of nanoparticles with proteins and how this interaction modifies the nanoparticles’ surface is crucial before their use for biomedical applications. Since fluorinated materials are emerging as potential imaging probes and delivery vehicles, their interaction with proteins of biological interest must be studied in order to be able to predict their performance in real scenarios. It is known that fluorinated planar surfaces may repel the unspecific adsorption of proteins but little is known regarding the same process on fluorinated nanoparticles due to the scarce examples in the literature. In this context, the aim of this work is to propose a simple and fast methodology to study fluorinated nanoparticle-protein interactions based on interfacial surface tension (IFT) measurements. This technique is particularly interesting for fluorinated nanoparticles due to their increased hydrophobicity. Our study is based on the determination of IFT variations due to the interaction of quantum dots of ca. 5 nm inorganic core/shell diameter coated with fluorinated ligands (QD_F) with several proteins at the oil/water interface. Based on the results, we conclude that the presence of QD_F do not disrupt protein spontaneous film formation at the oil/water interface. Even if at very low concentrations of proteins the film formation in the presence of QD_F shows a slower rate, the final interfacial tension reached is similar to that obtained in the absence of QD_F. The differential behaviour of the studied proteins (bovine serum albumin, fibrinogen and apotransferrin) has been discussed on the basis of the adsorption affinity of each protein towards DCM/water interface and their different sizes. Additionally, it has been clearly demonstrated that the proposed methodology can serve as a complementary technique to other reported direct and indirect methods for the evaluation of nanoparticle-protein interactions at low protein concentrations.

## 1. Introduction

The interaction of colloidal nanoparticles (NPs) with proteins leading to the formation of the protein corona is highly relevant for the field of nanomedicine and nanotoxicity [1,2,3]. The study of this interaction is fundamental for the understanding of the behaviour and fate of nanomaterials in biological media [4]. This interaction is a dynamic process governed by the binding affinities and equilibrium constants of each type of protein to the respective NP surface and it is very much dependent on the physicochemical properties, in particular surface charge or hydrophobicity, as provided by the NPs’ coating [5]. The presence of proteins adsorbed onto NPs that are intended for a biomedical application may alter the stability, uptake or excretion profile of those NPs [6,7]. For these reasons, many researchers have focused their attention on the study of protein corona formation and avoidance [8] and, indeed, many different experimental strategies have been applied to evaluate protein adsorption [9]. The adsorbed proteins can be directly analysed, such as with mass spectroscopy, circular dichroism and so forth. Alternatively, also indirect analysis may be used via measuring changes in the properties of the NPs, such as changes in size, optical properties and so on. In both cases, some techniques allow for the detection of the protein corona in situ, while others require the removal of unbound excess proteins before measurements, which however may change equilibrium properties. Measurement of size is one of the most straight-forward and obvious indirect methods used to study the protein corona, based on the hydrodynamic size increase of the nanomaterial due to the presence of proteins adsorbed onto their surface [9].

Regarding the interaction of quantum dots (QDs) with biological material such as proteins [10] or cells [11] at air/water interfaces, extensive research works have been reported by using the Langmuir monolayer approach [12] and using surface pressure-area isotherms and in situ UV-vis absorption and fluorescence spectroscopy as characterization techniques. The use of QDs in combination with Langmuir monolayers, which serves as model membranes, can be beneficial when it comes to study neurodegenerative diseases such as Alzheimer’s disease. This is due to the fact that the amyloid beta polypeptide, the major constituent of the amyloid plaques observed in Alzheimer’s disease patients, is formed at the interface of the biomembrane [10]. Interestingly, it was found that the nature of the capping of QDs played an important role in their interaction with the amyloid beta polypeptide, being even able to inhibit the fibrillation process [10]. In the context of fluorinated compounds, on the one hand, it is reported that fluorinated small molecules are prone to interact with hydrophobic pockets present in proteins [13]. On the other hand, it is reported that highly fluorinated surfaces repel protein adsorption [14]. However, little is known on the same phenomenon on fluorinated curved surfaces like NPs. Previous work in our group suggests that proteins may adhere to fluorinated NPs under certain circumstances [15,16]. For instance, when the size of fluorinated PEGylated NPs was interrogated in the absence or presence of proteins, we did not detect an increase in size but either a decrease or no size changes were observed [15]. It must be taken into account that a decrease or no change of size maybe an indirect indication of protein adsorption that may provoke the compression of flexible PEG. However, when using shorter non-PEGylated fluorinated ligands grafted onto quantum dots (QDs) as example for fluorescence NPs, we were able to encapsulate enzymes taking advantage of fluorine interaction with the selected enzymes [16]. Hence, this could indicate that proteins in physiological media may adsorb onto fluorinated nanoparticles. Given the fact that the presence of fluorine increases the hydrophobicity of the NPs, we believe that the study of changes in the interfacial tension (IFT) of fluorinated QDs (QD_F) which are able to interact with enzymes, may be an easy and straightforward approach to detect the presence of NP-protein interactions. This technique is particularly interesting for fluorinated materials that are not water-soluble and hence, they cannot be measured by standard techniques in a homogenous aqueous mixture with proteins. Furthermore, IFT measurement by using the pendant drop method is a well-established technique to study the self-organization of proteins in the form of films at the oil/water or air/water interfaces [17,18,19]. In this work, we intend to study the changes in the dynamic interfacial tension of mixtures of selected proteins and QD_F in order to gain information on how the presence of these fluorinated NPs may alter the protein organization at the oil/water interface [20]. This method may complement conventional techniques, which are largely based on optical, spectroscopic, fluorescence polarization, chromatographic or atomic force microscopy measurements [9]. Note that this method allows for in situ measurements, without the need of removing the excess proteins and therefore the equilibrium properties are not altered. Herein, we have used this technique to study the interactions of QD_F in dichloromethane (DCM) with aqueous solutions of bovine serum albumin (BSA), fibrinogen (FIB) and apotransferrin (aTR) at the oil/water interface.

## 2. Materials and Methods

### 2.1. Synthesis of Fluorinated Quantum Dots

Fluorinated quantum dots (QD_F) were prepared by ligand-exchange of TOPO-capped core/shellCdSe/ZnS quantum dots with a fluorinated ligand. Hydrophobic trioctylphosphine oxide (TOPO)-capped CdSe/ZnS core-shell quantum dots (QDs) with a CdSe/ZnS inorganic core/shell diameter of ca. 5 nm were synthesized by the hot injection method, as reported previously [21]. The protocol uses CdO and selenium as precursors for the CdSe cores and the shell of ZnS is formed using diethyl zinc and hexamethyldisilathiane as zinc and sulphur sources, respectively. The used fluorinated ligand, HS-C_11_-(EG)_4_-O-C(CF_3_)_3_ (EG = ethyleneglycol), forming the organic shell around the inorganic core/shell NPs, was synthetized as previously reported [22].

For the ligand exchange, the QD_TOPO dispersed in dichloromethane (DCM) were first precipitated with methanol (MeOH) to remove potential free TOPO ligands desorbed from the surface during storage and were then redispersed in DCM. The fluorinated ligand dissolved in DCM (2.3 mL, 42.7 mM, 100 µmol) was added to a 7.5 mL DCM:MeOH (1:2) mixture in a glass vial. Next, the solution of QD_TOPO (5 nmol, 0.6 mL, 8.3 μM NP concentration as determined by inductively coupled plasma mass spectrometry (ICP-MS), see calculation in the Supplementary Materials) was added and the mixture stirred overnight in darkness at 40 °C. Afterwards the NPs were purified by centrifugation at 1.5 × 10^5^ RCF for 30 min. The supernatant containing unbound ligands was removed and the NP precipitate was redispersed in 0.5 mL of DCM, 3 mL of MeOH was added for precipitating the NPs and the sample was centrifuged again at 1.5 × 10^5^ RCF for 30 min. Three cycles of washing were carried out to remove completely all unbound ligands. After the last centrifugation step, the precipitate containing the purified QD_FNPs was dispersed in DCM and stored in the fridge (4 °C) in darkness until further use.

### 2.2. Characterization of Fluorinated Quantum Dots

The size and shape of the QD_FNPs were determined by transmission electron microscopy (TEM). TEM images were acquired with a JEOL JEM 2100F microscope by deposition of the sample on top of a copper grid coated with a layer of carbon. The free software ImageJ was used for the analysis of the mean diameter of the inorganic NP part and the corresponding size distribution. The histograms were plotted with the software Origin. The QD concentration was obtained from ICP-MS data.

The presence of fluorinated ligands on the QD_F surface was confirmed by ^1^H and ^19^F NMR spectra, which were recorded in CD_2_Cl_2_ solution (purchased from Cortecnet) in a Bruker 500 MHz spectrometer (500 MHz for ^1^H and 470 MHz for ^19^F, Bruker, Ettlingen, Germany). Spectra were analysed with the software MestReNova (version 10.0.2.-15465).

The absorption spectrum of the QD_F NPs was acquired with a Varian 5000 UV-Vis-NIR spectrophotometer using a 10 mm path length glass cuvette. The fluorescence emission spectrum was collected with a Fluorolog-3 (model FL3-22, Horiba, Kyoto, Japan) fluorescence spectrometer using a 10 mm path length quartz cuvette and excitation at 400 nm. 

### 2.3. Interfacial Tension Measurements

The dynamic interfacial tension (IFT) of the QD_FNPs at the dichloromethane-water interface was measured by the pendant drop technique using a DSA100 measuring instrument (Krüss, Hamburg, Germany) [23]. An ultrafast camera (Krüss, Hamburg, Germany) was used to image the time-dependent drop shape changes of a sample droplet of oil (DCM solution) immersed in the aqueous phase. A Hamilton syringe plugged to a stainless steel needle was used to dispense a drop of the sample (a solution of QD_F in DCM at 100 nM as determined by IPC-MS) into the aqueous phase (buffer solution at fixed pH values containing different amount of protein). The time-dependent drop shape changes from drop fall until equilibrium were imaged by an ultrafast automated camera (examples are shown in Figure S3). Each measurement was performed at least in triplicate. The interfacial tension (***γ**_t_*) was estimated by fitting the droplet profile with the Young-Laplace equation using the analysis software package DSA3 (Krüss, Hamburg, Germany). The droplet profile was recorded and fitted obtaining a time dependent variation of the interfacial tension (IFT curve). The mean values and the standard deviations of each interfacial tension measurement within the experimental replicas were plotted and fitted to the empirical Hua and Rosen equation [24], cf. Equation (1):

***γ**_t_* = ***γ**_m_* + (***γ***_0_ − ***γ**_m_*)/(1 + (*t*/t*)^*n*^) (1)

where ***γ**_t_* is the surface tension at any time *t*, ***γ***_0_ is the surface tension of the pure solvent, ***γ**_m_* is the surface tension at meso-equilibrium, t* is the half-time in reaching ***γ**_m_* and *n* is a dimensionless exponent. Assuming a constant value of ***γ***_0_ = 28 mN/m for the surface tension value of the dichloromethane-aqueous buffer solution interface, ***γ**_m_*, t* and *n* remain as fit parameters. These parameters were estimated by computer fitting using the Origin software (version 8.6.0).

## 3. Results and Discussion

**Figure 1:**
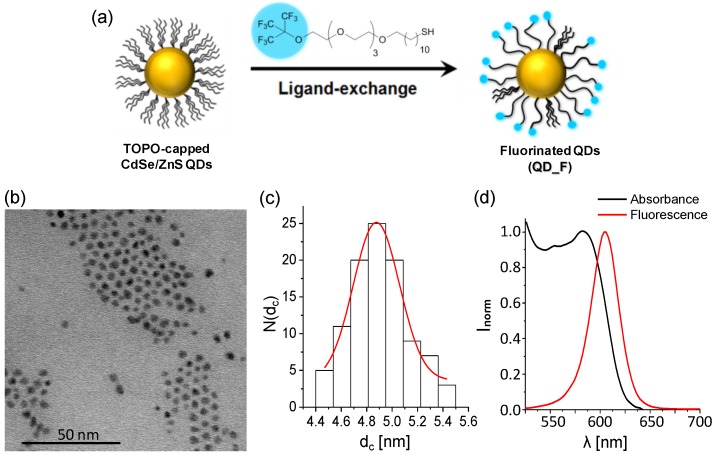
Synthesis and characterization of fluorinated quantum dot nanoparticles (QD_F NPs). (**a**) Scheme of ligand exchange on trioctylphosphine oxide (TOPO)-capped QDs using fluorinated ligands; (**b**) Transmission electron microscopy (TEM) image of QD_F NPs; (**c**) Histogram of the size distribution of the inorganic core/shell diameter (d_c_). N(d_c_) refers to the total counts; (**d**) Normalized absorption and fluorescence spectra of QD_F NPs in dichloromethane (DCM). Fluorescence emission was recorded under an excitation wavelength of 400 nm.

The preparation of the QD_F NPs was carried out by ligand-exchange of TOPO-capped CdSe/ZnS QDs with a fluorinated ligand (HS-C_11_-(EG)_4_-O-C(CF_3_)_3_). By ^1^H and ^19^F nuclear magnetic resonance (NMR) we could confirm the presence of fluorinated molecules and some remaining TOPO molecules that did not exchange (see Figure S1 in the Supplementary Materials). The amount of fluorinated ligand was sufficient to alter the hydrophobic features of the QD_F as compared to QD_TOPO, leading to a modification of the interfacial tension profile as shown in Figure S2 of the Supplementary Materials. As expected, the so-obtained QD_F were soluble in organic solvents such as dichloromethane (DCM) but not in water due to the hydrophobic character of both, TOPO and the fluorinated ligands. QD_F presented a size of (4.9 ± 0.4) nm in the inorganic CdSe/ZnS core/shell diameter d_c_ as determined by TEM and maximum emission fluorescence at 605 nm under excitation at 400 nm (Figure 1).

Fluorinated QDs were selected owing to their interest as combined delivery vehicles and fluorescence imaging probes [13]. In addition, and as mentioned before, our previous studies regarding enzyme encapsulation in QD_F nanoassemblies clearly suggest that fluorinated heads have an attraction for protein-like molecules [13]. Since these QD_F are not water-soluble, the study of their interactions with proteins directly in aqueous media is not possible, for which IFT is herein suggested as an indirect method to observe these types of interactions.

Hence, the pendant drop technique was used to interrogate the IFT profile of QD_F solutions in the presence of increasing concentrations of three selected proteins, which are commonly found in physiological media, that is, BSA, FIB and aTR. A drop of a dichloromethane solution of QD_F (100 nM) was formed in aqueous solutions in the presence or absence of proteins and the dynamic interfacial tension (***γ**_t_*) measured over 1000 s until a plateau was observed. The concentration of QD_F (100 nM) was selected taking into account our previous studies with enzymes, in which that amount of QDs was optimal for interaction [13]. To avoid differences based on electrostatic forces, all measurements were performed at a pH corresponding to the isoelectric point of each protein (pH = 5, 5.5 and 6.1 for BSA, FIB and aTR, respectively). Britton-Robinson buffer solutions (“universal” pH buffer) were used with the aim of maintaining the same buffer composition in the whole range of pH.

**Figure 2:**
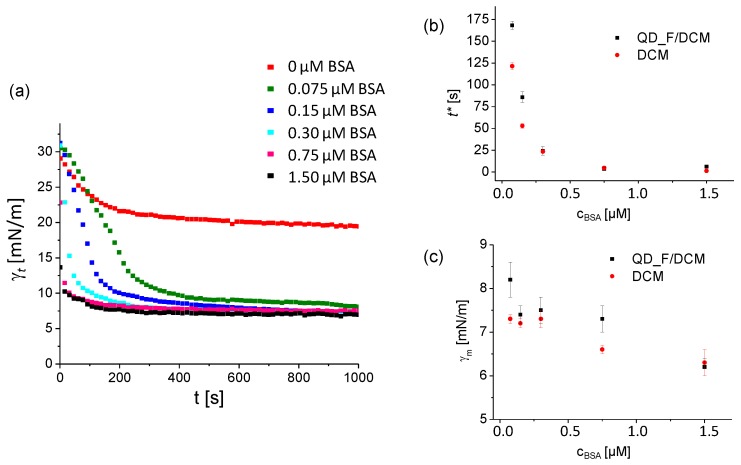
(**a**) Time dependence of the interfacial tension (IFT) ***γ**_t_* for QD_F (100 nM in DCM) immersed in aqueous buffer solutions at pH 5 containing increasing concentrations of bovine serum albumin (BSA) (from 0 to 1.5 µM). Measurements were performed in triplicate; (**b**) Plot of t* versus BSA concentration c_BSA_ of either DCM alone or QD_F (100 nM) in DCM; (**c**) Plot of ***γ**_m_* versus BSA concentration c_BSA_ of either DCM alone or QD_F (100 nM) in DCM.

Initially, BSA was selected as a model protein since its adsorption in the form of films at the DCM/water interface was already reported [14,15,16]. The IFT profile of a DCM drop (without QD_F) immersed in aqueous solutions of increasing BSA concentration was measured and compared with the IFT profile of QD_F in DCM exposed to the same BSA concentrations at pH = 5 (Figure 2a). As the BSA amount increased, the interfacial tension decreased due to BSA film formation at the oil/water interface, as expected. The same trend was observed both, in the presence and absence of QD_F NPs. Indeed, the obtained ***γ**_m_* values under both experimental settings were very similar, as shown in Figure 2c. On the contrary, the t*, that is, the time that the system needs to reach meso-equilibrium was affected by the presence of QD_F for very diluted samples of BSA. Interestingly, the presence of QD_F in the oil phase induced larger t* values for BSA concentrations below 0.3 µM, suggesting that the presence of QD_F NPs slowed down the BSA adsorption at the oil/water interface. However, at BSA concentrations greater than 0.3 µM, the presence of QD_F did not alter the BSA film formation kinetics (Figure 2b).

**Figure 3:**
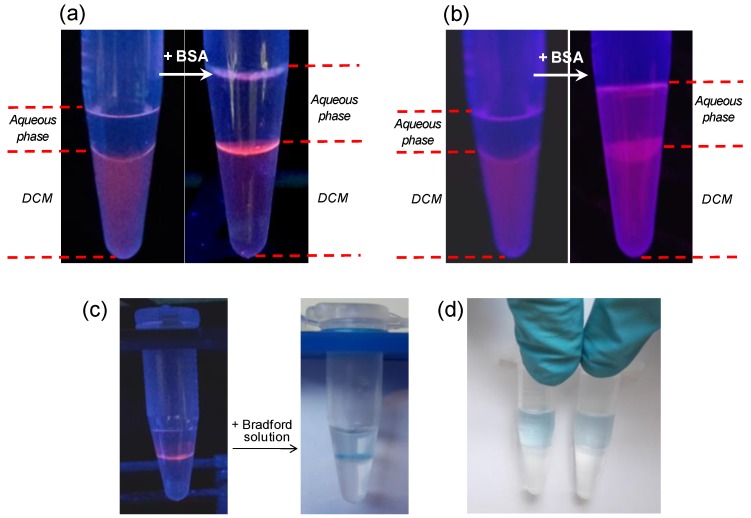
(**a**) Photographs under UV lamp before and after the interaction of the BSA protein as dissolved in a buffer solution at pH 5 with a solution of QD_F in DCM, showing the self-assembly of the NPs at the oil/water interface after BSA interaction; (**b**) Control experiment with QD_TOPO showing that the NPs are finally randomly distributed; (**c**) Photograph of the Bradford reagent developing at the interface when BSA was exposed to QD_F solution; (**d**) Control experiment of Bradford reagent developing homogeneously in the aqueous phase containing BSA (1.5 µM on the left and 0.075 µM on the right) and DCM without QD_F as the oil phase.

The location of the QD_F could be easily followed with an UV lamp thanks to the fluorescence of the QDs. In the absence of BSA, QD_F were distributed homogeneously in the DCM phase. On the contrary, when BSA was present, a thin layer of QD_F could be clearly observed at the oil/water interface (Figure 3a). A control experiment with TOPO-capped QDs (QD_TOPO), that is QDs coated also with hydrophobic ligands but non-fluorinated, did not lead to the same result (Figure 3b). In this case the QDs were finally distributed randomly between the organic and aqueous phases as well as at the interface after their interaction with the BSA, showing thus a non-preferential adsorption at the oil/water interface as observed previously with the QD_F NPs. This fact may indicate that the fluorinated head −C(CF_3_)_3_ in the QD_F could play a major role in the self-assembly of these QDs at the oil/water interface in the presence of proteins. Moreover and in order to visualize the final location of the BSA protein in the former example, a few drops of Bradford reagent were added into the aqueous phase and left for some minutes. The developing of the blue colour mostly at the oil/water interface indicated that the majority of the protein was at the interface interacting with the QD_F (Figure 3c). Surprisingly this QD_F-protein interaction was stable and remained unchanged for long time (more than 2 weeks). On the contrary, when the same experiment was performed with DCM/BSA mixtures in the absence of QD_F NPs, the Bradford reagent spread homogenously into the aqueous phase (Figure 3d).

**Figure 4:**
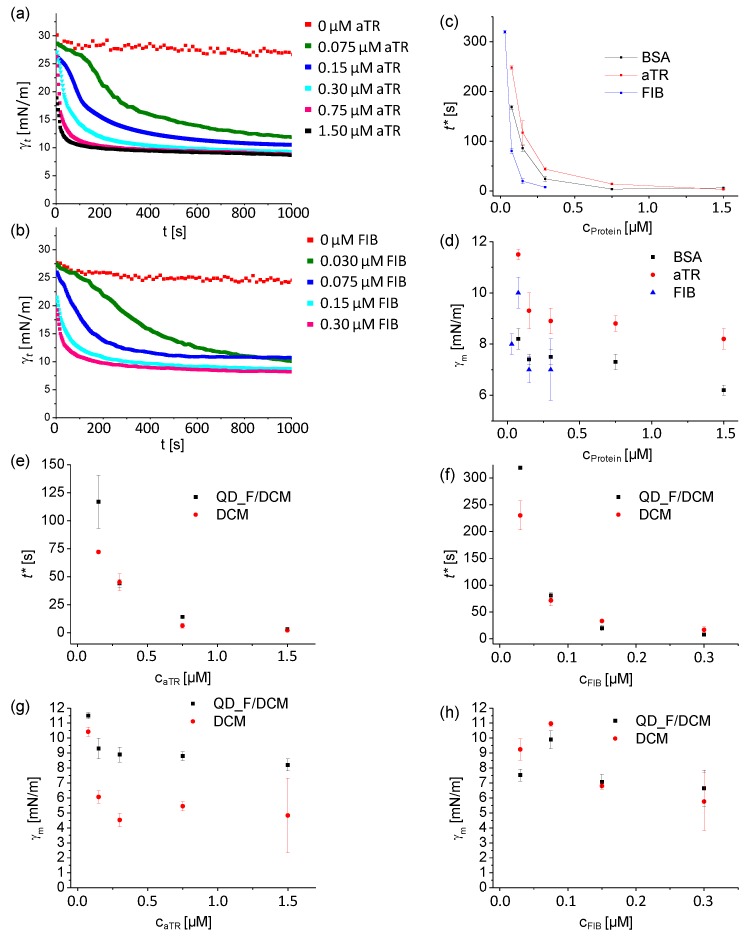
(**a**) Time dependence of the interfacial tension (IFT) ***γ**_t_* for QD_F (100 nM in DCM) immersed in aqueous buffer solutions at pH 6.1 containing increasing concentrations of apotransferrin (aTR) (from 0 to 1.5 µM). Measurements were performed in triplicate; (**b**) Time dependence of the interfacial tension (IFT) ***γ**_t_* for QD_F (100 nM in DCM) immersed in aqueous buffer solutions at pH 5.5 containing increasing concentrations of fibrinogen (FIB) (c_FIB_ from 0 to 0.3 µM). Measurements were performed in triplicate; (**c**) Plot of t* versus protein concentration c_protein_ of QD_F (100 nM) in DCM for BSA, aTR and FIB; (**d**) Plot of ***γ**_m_* versus protein concentration c_protein_ of QD_F (100 nM) in DCM for BSA, aTR and FIB; Plot of t* versus aTR concentration c_aTR_; (**e**) or FIB concentration c_FIB_; (**f**) of either DCM alone or QD_F (100 nM) in DCM; Plot of ***γ**_m_* versus aTR concentration c_aTR_ (**g**) or FIB concentration c_FIB_ (**h**) of either DCM alone or QD_F (100 nM) in DCM.

In addition, we ran additional measurements with other interesting proteins such as aTR and FIB (at pH = 6.1 and 5.5, respectively). In all cases we observed a similar trend, that is, a decrease in the interfacial tension in the presence of those proteins, indicating that aTR and FIB also self-assembly themselves at the oil/water interface in the presence of QD_F NPs (Figure 4a,b). Interestingly, we observed that each protein displayed a distinctive IFT profile. aTR displayed the highest ***γ**_m_* values of all three proteins and BSA and FIB had more similar values (Figure 4d, Table 1).

All dynamic interfacial tension curves (***γ**_t_* versus time) were well fitted with correlation coefficients ≥0.9 to the Hua and Rosen equation, obtaining the fitted parameters summarized in Table 1.

As shown in Figure 4d, there was a clear decay in the interfacial tension as the protein concentration increased. In the case of FIB, this effect was noticeable at much lower concentrations (Figure 4b), while aTR behaved quite similarly to BSA (Figure 4a vs. Figure 3a). These differences might be related to the adsorption affinity of each protein towards DCM/water interface. We do not have data regarding such adsorption affinities but Young et al., [25] reported that FIB displayed a much higher adsorption affinity towards polymeric surfaces than BSA and transferrin (aTR bound to iron), both of which had a similar behaviour. These data may help to explain the similarities in the behaviour of BSA and aTR in terms of protein concentration versus ***γ**_m_* and the differences of those with FIB. Likewise, and as observed for BSA, the t* of aTR and FIB was affected by the presence of QD_F in the oil phase at low concentrations of proteins. Again, the time required to achieve meso-equilibrium (t*) was longer when QD_F were present, than when only DCM was used as the oil phase (Figure 4e–h, Table 1). Pronounced differences were observed when comparing t* of all proteins. BSA reached the meso-equilibrium situation faster than aTR at equal concentration of protein, both in the absence and presence of QD_F (Figure 4c and Table 1). This fact may be related to the diffusion of the proteins in the water phase towards the oil/water interface, which is directly affected by the size of the protein. Hence, BSA, being slightly smaller than aTR (66 and 78 kDa, respectively) would diffuse faster and reach meso-equilibrium earlier than aTR (Figure 4c and Table 1). On the contrary, FIB (340 kDa in molecular weight) is of much greater size than BSA or aTR, for which it would, in principle, diffuse slower but would potentially need much less protein molecules to fill the oil/water interface than the other two proteins. This could be the reason behind the shorter t* values of FIB for the same protein concentration as BSA or aTR (Table 1).

In summary, preliminary results presented herein suggest that the presence of fluorinated quantum dots (QD_F) do not disrupt protein (BSA, aTR or FIB) spontaneous film formation at the oil/water interface, which may be taken as an indication of potential QD_F-protein interactions at such interface. At very low concentrations of proteins, the film formation in the presence of QD_F seems to achieve equilibrium at slower rate than in only DCM but the final interfacial tension ***γ**_m_* reached is similar to that obtained in the absence of QD_F. This conclusion was reached by IFT analysis of DCM solutions of QD_F in the presence of increasing concentrations of BSA, aTR or FIB at controlled pH. Hence, we propose IFT as a simple, fast and reproducible technique to evaluate NP-protein interactions, particularly for NPs that are not water-soluble and cannot be mixed homogenously with proteins for standard measurements. Interestingly, photographs taken under UV light in the presence of BSA and QD_F suggest that BSA triggered the self-assembly of fluorinated QDs at the interface. Further insight into this phenomenon is required to fully understand the scope and driving forces behind the self-assembly of QD_F at oil/water interfaces mediated by proteins.

## Figures and Tables

**Table 1:** Dynamic surface tension parameters calculated from kinetic curves (***γ**_t_* vs. time) of the adsorption of fluorinated quantum dots (QD_F) at the DCM/water interface under different protein amounts. Measurements are performed in triplicate and data are expressed as mean value ± standard deviation (SD).

**c_BSA_ (μM)**	**Oil Phase**	***γ_m_* (mN/m)**	t* (s)	**c_BSA_ (μM)**	**Oil Phase**	***γ_m_* (mN/m)**	t* (s)
0.075	DCM	7.4 ± 0.1	121.4 ± 3.9	0.075	QD_F	8.2 ± 0.4	168.2 ± 4.5
0.15	DCM	7.2 ± 0.1	52.9 ± 2.5	0.15	QD_F	7.4 ± 0.2	85.9 ± 6.2
0.3	DCM	7.3 ± 0.2	23.4 ± 0.7	0.3	QD_F	7.5 ± 0.3	24.2 ± 4.8
0.75	DCM	6.6 ± 0.1	4.7 ± 2.1	0.75	QD_F	7.3 ± 0.3	3.8 ± 1.5
1.5	DCM	6.3 ± 0.3	1.3 ± 0.2	1.5	QD_F	6.2 ± 0.2	0.7 ± 0.2
**c_aTR_ (μM)**	**Oil Phase**	***γ_m_* (mN/m)**	t* (s)	**c_aTR_ (μM)**	**Oil Phase**	***γ_m_* (mN/m)**	t* (s)
0.075	DCM	10.4 ± 0.3	197.5 ± 8.8	0.075	QD_F	11.5 ± 0.2	247.7 ± 3.6
0.15	DCM	6.1 ± 0.4	72.1 ± 1.5	0.15	QD_F	9.3 ± 0.7	116.9 ± 23.7
0.3	DCM	4.5 ± 0.4	45.1 ± 7.5	0.3	QD_F	8.9 ± 0.5	44.0 ± 3.6
0.75	DCM	5.5 ± 0.3	6.3 ± 2.2	0.75	QD_F	8.8 ± 0.3	14.0 ± 1.1
1.5	DCM	4.8 ± 2.5	2.2 ± 0.7	1.5	QD_F	8.2 ± 0.4	3.2 ± 0.6
**c_FIB_ (μM)**	**Oil Phase**	***γ_m_* (mN/m)**	t* (s)	**c_FIB_ (μM)**	**Oil Phase**	***γ_m_* (mN/m)**	t* (s)
0.03	DCM	9.2 ± 0.7	230.2 ± 27.0	0.03	QD_F	7.5 ± 0.4	319.3 ± 2.7
0.075	DCM	10.9 ± 0.2	71.2 ± 9.8	0.075	QD_F	9.9 ± 0.6	80.6 ± 4.8
0.15	DCM	6.8 ± 0.2	32.9 ± 1.0	0.15	QD_F	7.1 ± 0.4	19.6 ± 5.3
0.3	DCM	5.8 ± 1.9	16.3 ± 5.6	0.3	QD_F	6.7 ± 1.2	7.5 ± 1.4

## Supplementary Materials

The following are available online at http://www.mdpi.com/1996-1944/11/5/750/s1, Figure S1: (**a**) ^19^F NMR spectrum and (**b**) ^1^H NMR spectrum of QD_F, both recorded in CD_2_Cl_2_, Figure S2: Time dependence of the interfacial tension (IFT) ***γ**_t_* for QD_F (100 nM in DCM) and QD_TOPO (100 nM in DCM) immersed in aqueous buffer solution at pH 5 in the absence of proteins. Measurements were performed in triplicate, Figure S3: Example images of the change of the drop sample with the NPs (QD_F NPs at 100 nM in DCM) immersed in aqueous buffer solutions at pH 5 with different BSA concentrations from the initial time (*t* = 0) to the final measured time (*t* = 1000 s) when the meso-equilibrium is reached. The diameter of the Hamilton needle is 1.8 mm (stainless steel NE45; Krüss-scientific).
