# Supplementary material for: Study of Fluorinated Quantum Dots-Protein Interactions at the Oil/Water Interface by Interfacial Surface Tension Changes
Source: Materials (Basel). 2018 May 8;11(5):750. doi: 10.3390/ma11050750 (PMC5978127; doi:10.3390/ma11050750)
Supplement: Supplementary file 1 [file materials-11-00750-s001.pdf]

Supplementary Materials

Study of Fluorinated Quantum Dots-Protein Interactions at the Oil/Water Interface by Interfacial Surface Tension Changes

Carolina Carrillo-Carrión [1,2,*], Marta Gallego [2], Wolfgang J. Parak[2,3] and Mónica Carril[2,4]

[1] Centro Singular de Investigación en Química Biológica y Materiales Moleculares (CiQUS) y Departamento de Física de Partículas, Universidad de Santiago de Compostela, 15782 Santiago de Compostela, Spain
[2] Bioengineered Particles Group, CIC biomaGUNE, 20014 San Sebastian, Spain; mgallego@cicbiomagune.es (M.G.); wolfgang.parak@uni-hamburg.de (W.J.P.); mcarril@cicbiomagune.es (M.C.)
[3] Fachbereich Physik and CHyN, Universität Hamburg, 22607 Hamburg, Germany
[4] Ikerbasque, Basque Foundation for Science, 48011 Bilbao, Spain
* Correspondence: carolina.carrillo@usc.es; Tel.: +34-881-815-770

I. Additional Figures

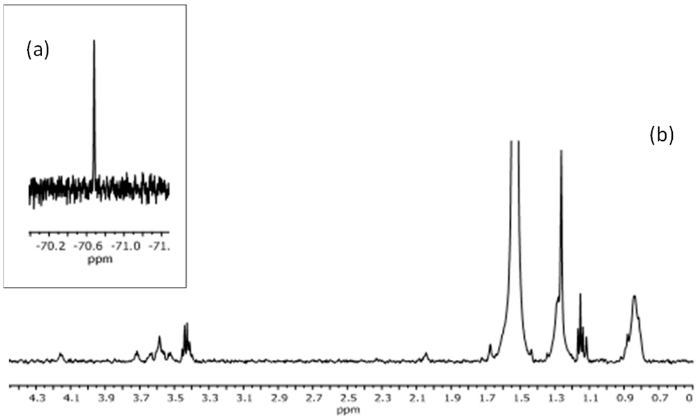

Figure S1. (**a**)^{19}F NMR spectrum and (**b**) ^{1}H NMR spectrum of QD_F, both recorded in CD_2Cl_2.

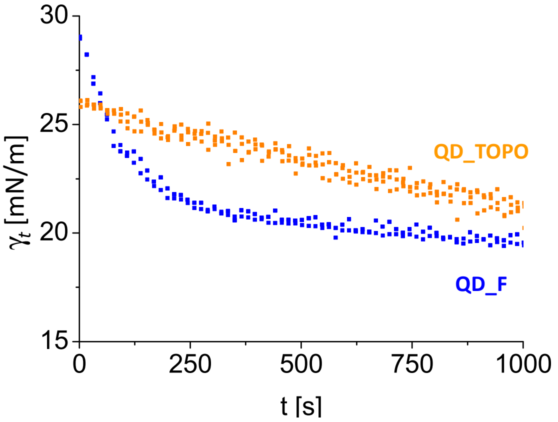

Figure S2. Time dependence of the interfacial tension (IFT) γ_t for QD_F (100 nM in DCM) and QD_TOPO (100 nM in DCM) immersed in aqueous buffer solution at pH 5 in the absence of proteins. Measurements were performed in triplicate.

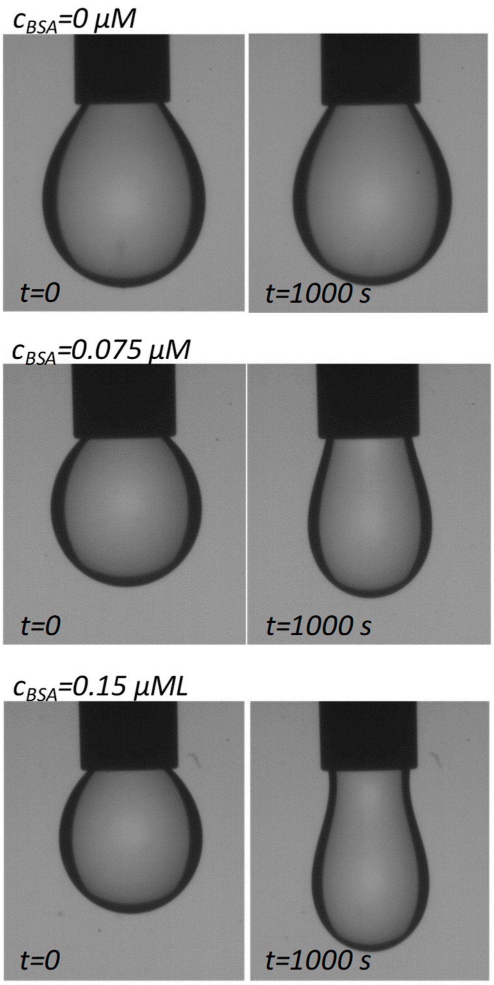

Figure S3._Example images of the change of the drop sample with the NPs (QD_F NPs at 100 nM in DCM) immersed in aqueous buffer solutions at pH 5 with different BSA concentrations from the initial time (t=0) to the final measured time (t=1000 s) when the meso-equilibrium is reached. The diameter of the Hamilton needle is 1.8 mm (stainless steel NE45; Krüss-scientific).

II. Calculation of the concentration of CdSe/ZnSQDs from ICP-MS data

Quantitative elemental analysis of the CdSe/ZnS nanoparticles was performed by inductively coupled plasma mass spectrometry (ICP-MS) after digestion of the sample with aqua regia (i.e., concentrated HCl (35 wt%) and HNO3 (67 wt%) in 3:1 volume ratio). Before the analysis of the sample, a proper calibration was performed using standards of the elements to be measured, which are Cd, Se, and Zn in the case of CdSe/ZnS nanoparticles. Note that due to the difficulty to determine S in a reliable a sensitive way we assumed that the ratio Zn:S is 1:1.

The data obtained from ICP-MS analysis are the mass of each element in the NP solution of volume V (m_{Cd}, m_{Se}, m_{Zn}). Taking into account that the molar stoichiometry Zn:S is assumed to be 1:1 in the NP, the mass of elemental sulphur in solution can be calculated as:

$m_S=(m_{Zn}\cdot M_S)/M_{Zn}$

Hereby M_S and M_{Zn} are the atomic weight of sulphur and zinc, respectively.

On the one hand, the mass of CdSe cores in solution is:

$m_{CdSe} = m_{Cd} + m_{Se}$

Using the density of CdSe ($\rho_{CdSe} = 5.82$ g/cm^3) and the mass of CdSe in solution, the volume of all the CdSe cores (V_{CdSe}) in solution can be calculated as:

$V_{CdSe} = m_{CdSe} / \rho_{CdSe}$

On the other hand, the mass of ZnS (i.e. shell of the NP) in solution is:

$m_{ZnS} = m_{Zn}+ m_S$

In the same way, using the density of ZnS ($\rho_{ZnS} = 4.09$ g/cm^3) and the mass of ZnS in solution, the volume of the ZnS shell (V_{ZnS}) in solution can be calculated as:

$V_{ZnS} = m_{ZnS} / \rho_{ZnS}$

Thus, the total volume of all the core/shell NPs ($V_{t,NP}$) is:

$V_{t,NP} = V_{CdSe} + V_{ZnS}$

Considering an average NP diameter of $d_c = 4.9$ nm as obtained by TEM and taking into account the spherical shape of the core/shell CdSe/ZnS NPs, the volume of one NP (V_{NP}) with inorganic core/shell (CdSe/ZnS) diameter $d_c$is:

$V_{NP}= 4/3\cdot\pi\cdot(d_c/2)^3$

Knowing the total volume of NPs in solution ($V_{t,NP}$) and the volume of one NP (V_{NP}), the total number of NPs in solution (N_{NP}) can be calculated as:

$N_{NP}= V_{t,NP}/V_{NP}$

Then, the molar concentration of CdSe/ZnS NPs (N_{NP}) in the solution of volume V is determined as:

$c_{NP} = N_{NP}/N_A/V$

Hereby N_A is the Avogadro constant.Note that in this volume based-calculation the core/shell geometry is not included, which due to the different densities of CdSe and ZnS introduces some error.
